# Cell Wall Enzymes in *Zygnema circumcarinatum* UTEX 1559 Respond to Osmotic Stress in a Plant-Like Fashion

**DOI:** 10.3389/fpls.2019.00732

**Published:** 2019-06-07

**Authors:** Elisabeth Fitzek, Lauren Orton, Sarah Entwistle, W. Scott Grayburn, Catherine Ausland, Melvin R. Duvall, Yanbin Yin

**Affiliations:** ^1^Department of Biological Sciences, Plant Molecular and Bioinformatics Center, Northern Illinois University, DeKalb, IL, United States; ^2^Department of Computational Biology, Bielefeld University, Bielefeld, Germany; ^3^Center for Biotechnology, Bielefeld, Germany; ^4^Department of Food Science and Technology, Nebraska Food for Health Center, University of Nebraska – Lincoln, Lincoln, NE, United States

**Keywords:** charophyte green algae, RNA-seq, *Zygnema circumcarinatum*, glycosyltransferases, osmotic stress, gene expression

## Abstract

Previous analysis of charophyte green algal (CGA) genomes and transcriptomes for specific protein families revealed that numerous land plant characteristics had already evolved in CGA. In this study, we have sequenced and assembled the transcriptome of *Zygnema circumcarinatum* UTEX 1559, and combined its predicted protein sequences with those of 13 additional species [five embryophytes (Emb), eight charophytes (Cha), and two chlorophytes (Chl) as the outgroup] for a comprehensive comparative genomics analysis. In total 25,485 orthologous gene clusters (OGCs, equivalent to protein families) of the 14 species were classified into nine OGC groups. For example, the Cha+Emb group contains 4,174 OGCs found in both Cha and Emb but not Chl species, representing protein families that have evolved in the common ancestor of Cha and Emb. Different OGC groups were subjected to a Gene Ontology (GO) enrichment analysis with the Chl+Cha+Emb group (including 5,031 OGCs found in Chl and Cha and Emb) as the control. Interestingly, nine of the 20 top enriched GO terms in the Cha+Emb group are cell wall-related, such as biological processes involving celluloses, pectins, lignins, and xyloglucans. Furthermore, three glycosyltransferase families (GT2, 8, 43) were selected for in-depth phylogenetic analyses, which confirmed their presence in UTEX 1559. More importantly, of different CGA groups, only Zygnematophyceae has land plant cellulose synthase (CesA) orthologs, while other charophyte CesAs form a CGA-specific CesA-like (Csl) subfamily (likely also carries cellulose synthesis activity). Quantitative real-time-PCR experiments were performed on selected GT family genes in UTEX 1559. After osmotic stress treatment, significantly elevated expression was found for GT2 family genes ZcCesA, ZcCslC and ZcCslA-like (possibly mannan and xyloglucan synthases, respectively), as well as for GT8 family genes (possibly pectin synthases). All these suggest that the UTEX 1559 cell wall polysaccharide synthesis-related genes respond to osmotic stress in a manner that is similar to land plants.

## Introduction

Recently charophytes received much attention while studying the terrestrialization of land plants (McCourt et al., 2004; Timme and Delwiche, 2010; Heidel et al., 2011; Delwiche and Cooper, 2015; Bowman et al., 2017; de Vries and Archibald, 2018; Nishiyama et al., 2018). Charophyte green algal (CGA) consist of 122 genera with more than 10,000 species and are predominantly found in freshwater habitats (McCourt et al., 2004; Delwiche and Cooper, 2015; Domozych et al., 2016). In terms of taxonomy, CGA can be divided into six classes of two clades (i) KCM-clade: basal charophytes (Klebsormidiophyceae, Chlorokybophyceae, and Mesostigmatophyceae) and (ii) ZCC-clade: later evolved charophytes, more closely related to land plants (Zygnematophyceae, Coleochaetophyceae, and Charophyceae) (de Vries et al., 2016). Within ZCC, Zygnematophyceae are highly diverse and the largest group of CGA (Delwiche and Cooper, 2015; Domozych et al., 2016). Furthermore, phylogeny analysis has placed the Zygnematophyceae class as the closest sister group to land plants (Wickett et al., 2014; Delwiche and Cooper, 2015).

Compared to other CGA such as *Nitella* of the Charophyceae, Zygnematophyceae has a simpler body plan as unicellular and unbranched filamentous algae. Delwiche and Cooper (2015) stated that, for the ancestor of land plants, it was more relevant to cope with hydrological gradient than the presence of a branching body type. For example, we found that the Zygnematophyceae species *Zygnema circumcarinatum* produces an abundant amount of mucilage, which is advantageous to retain water and protect from desiccation, potentially a key feature for the ancestral algae to adapt to the wet-to-dry transition (Becker and Marin, 2009; Timme and Delwiche, 2010).

Currently, only two CGA nuclear genomes are available: the basal Klebsormidiophyceae species, *Klebsormidium nitens* NIES-2285 (previously known as *K. flaccidum*), and the later branching Charophyceae species, *Chara braunii* (Hori et al., 2014; Nishiyama et al., 2018). However, numerous RNA sequencing transcriptomes are available, which are often easier to assemble and analyze. These RNA-Seq data also provided valuable resources toward the study of algal response physiology (e.g., cold, high light, drought, phytohormone). Recently, new research has added to the pool of transcriptome data available for each representative within the CGA clade (Holzinger et al., 2014; Hori et al., 2014; Ju et al., 2015; Van de Poel et al., 2016; Rippin et al., 2017; de Vries et al., 2018). Special interest was directed toward members of the ZCC clade, such as *Spirogyra pratensis* and *Z. circumcarinatum*. Comparative analysis across the plant kingdom revealed orthologs for plant hormone biosynthesis and signaling, the NDH (NADPH dehydrogenase) complex, and phytochromes, in different ZCC species. These provided valuable insight into the understanding of evolutionary adaptations that occurred during early plant terrestrialization and significantly improved the knowledge of land plant evolution.

Here, we present the assembled and annotated transcriptome of *Z. circumcarinatum* strain UTEX 1559. Two recent studies have published RNA-Seq data of other strains of *Z. circumcarinatum*: (i) strain SAG 698-1a was studied in a comparative transcriptome analysis focusing on the response to cold and high light stresses across six CGA classes (de Vries et al., 2018); (ii) strain SAG 2419 was used to study the response to a year-long dehydration and desiccation tolerance using both liquid and plate cultures (Rippin et al., 2017). We noted that SAG 698-1a was just one of the six species studied in the first paper, and SAG 2419 studied in the second paper was reported to have contaminated RNAs from bacteria and other eukaryotes.

Our UTEX 1559 axenic culture is derived from *Z. circumcarinatum* 42PE strain, which was purified by Gauch (1966). Different from previous studies, our goals in this study were to: (i) analyze the transcriptome of UTEX 1559 and compare the gene contents of 14 plant and algal genomes/transcriptomes to identify gene functional groups that only exist in CGA and land plants, and (ii) identify key genes for cell wall synthesis in UTEX 1559, in order to better understand plant cell wall evolution in relation to plant adaptation to terrestrial environment. Expression of 15 selected UTEX 1559 cell wall-related genes were measured with qRT-PCR quantification in respect to osmotic stress.

With respect to the second goal, polysaccharides such as celluloses, hemicelluloses (containing four classes: xylans, mannans, xyloglucans, and mixed-linkage glucans or MLGs), and pectins, are major building components of plant and algal cell walls (Sørensen et al., 2011). They provide protection and stability to land plants and algae (Popper and Tuohy, 2010). These building components are synthesized by different glycosyltransferases (GT) families of carbohydrate-active enzymes (CAZymes). Recent phylogenetic studies have revealed the presence of cellulose synthases (CesAs) and hemicellulose synthases (CesA-like or Csl) of GT2, as well as xylan and pectin synthesis related GT8 and GT43 in early eukaryotes (Yin et al., 2009, 2010; Taujale and Yin, 2015). Interestingly, CGA have representatives within CesA, CslC, CslD, and CslK subfamilies (Mikkelsen et al., 2014; Yin et al., 2014). GT43 consists of three clades, namely A, B, and C, of which the latter was suggested to have evolved the earliest in CGA (Taujale and Yin, 2015). As for GT8, previous analyses have found CGA representatives having the cell wall synthesis group, such as α-galacturonosyltransferase 1 and 12 (GAUT1, GAUT12) and galacturonosyltransferase-like (GATL), as well as the starch synthesis group, plant glycogenin-like starch initiation proteins (PGSIPs) of the GT8 family (Mikkelsen et al., 2014). As mentioned above, we aimed to identify these genes in UTEX 1559 transcriptome and verify their expression using qRT-PCR in relation to osmotic stress.

## Materials and Methods

### *Zygnema circumcarinatum* UTEX 1559 Growth Conditions, Harvest and RNA Extraction

UTEX 1559 algae were purchased from utex.org (University of Texas at Austin’s Culture Collection of Algae). A few filaments were transferred to 50 ml liquid culture media, Bold’s basal media (BBM) or modified Bold’s basal media (MBBM), and grown for 3–4 weeks on a rotary shaker (Fermentation Design, 150 rpm) in a Conviron PGW36 growth chamber (110 μmol m^−2^ s^−1^, 16/8 of light/dark cycle, 28°C). To obtain a wide range of expressed genes, different carbon sources were added to separate media. Glucose (0.5% w/v), cellobiose (0.5% w/v), yeast extract and glucose (0.04% w/v) with glucose (0.5% w/v), or yeast extract (0.04% w/v) and cellobiose (0.5% w/v) were added separately to BBM to obtain different MBBMs. The algal cultures were harvested using a vacuum regulator (Bio-Rad, 7 in. Hg) with a filter system (Nalgene) using autoclaved Whatman filter paper (#2 qualitative). The algae were transferred to 1.5 ml sterile Eppendorf tubes. Fresh weight was measured and algae were stored at −80°C. Frozen algae were subjected to 16–22 h of lyophilization VirTis Sentry 2.0, at −50°C). The lyophilized algae were ground to a fine powder for 8 min using sterile pestles and sterile metal spatulas. RNA was extracted using NucleoSpin plant II kit (Machery-Nagel, Germany) following the manufactures protocol using lysis buffer PL2. Total RNA was isolated from an average of 778 mg (fresh weight) per sample. To determine the integrity and presence of 18S and 28S rRNA, 5–10 μl of the purified RNA was loaded onto 1% (w/v) agarose gel in tris acetate EDTA buffer.

### RNA Sequencing and Assembly

RNA samples were shipped on dry ice to Roy J. Carver Biotechnology Center at University of Illinois at Urbana-Champaign. Library preparation using the TruSeq Standard RNA-Seq sample prep kit (Illumina, San Diego, CA, United States), and 260 bp paired-end sequencing via the HiSeq 2500 system were performed. Read quality was assessed using FASTQC v. 3 (Andrews, 2010). Low quality and low complexity reads were removed with prinseq-lite −0.20.4 (Schmieder and Edwards, 2011). All reads were paired-end, and assembled using Trinity version 2.1.1, release 2012-06-01 (Grabherr et al., 2013).

### RNA-Seq Annotation

The longest open reading frame (ORF) of each assembled transcript/contig was predicted using the software TransDecoder, part of the Trinity package (Grabherr et al., 2013). Potential homologs were identified using BLASTP and BLASTX against *K. nitens*, *Arabidopsis thaliana*, and Swiss-Prot (Table 1). The *A. thaliana* protein database was downloaded from The Arabidopsis Information Resource (TAIR 10^1^). The *K. nitens* protein database was downloaded from http://www.plantmorphogenesis.bio.titech.ac.jp/∼algae_genome_project/klebsormidium/kf_download.htm. The *E*-value threshold was set to 1e-5. Protein domain identification (Pfam) was performed using hmmscan version 3.1b1 (Finn et al., 2015) with *E*-value cut off 1e-5. Trinity contigs, and TransDecoder ORFs, top BLAST hits, and hmmscan hits were together uploaded into a SQL-lite database to generate an annotation file using the Trinotate pipeline (Grabherr et al., 2013).

**Table 1:** Summary of UTEX 1559 RNA-Seq assembly and annotation.

Product	Count	Percent
Total^#^ of genes	58,087	
Total^#^ of transcripts	66,952	
Total^#^ of proteins	43,573	
GC%	50.6	
Contig N50	2,011 bp	
Avg. contig length	1,027 bp	
Total assembled bases	68,772,302	
TAIR10_BLASTX	25,366	37.88^a^
Kni_BLASTX	27,166	40.57^a^
UniRef_ BLASTP	29,133	66.86^b^
TAIR10_BLASTP	24,608	56.48^b^
Kni_BLASTP	26,516	60.86^b^

*^a^Calculated in respect to the number of transcripts (66,952). ^b^Calculated in respect to the number of proteins (43,573).*

### Gene Family Analysis

TransDecoder predicted proteins of UTEX 1559 were combined with proteins of 13 other species selected from a variety of plant/algal taxonomic groups (see Table 2 for the list of species, the source of data, and the number of proteins in each species). Among the total 14 species, six CGA species (including UTEX 1559) only have transcriptomes available. For these six species, we used the same RNA-Seq assembly, protein prediction, and annotation protocol as described above for UTEX 1559.

**Table 2:** List of species and their protein count.

Clade	Species	Abbreviation	Source	Number of proteins
*Embryophyte (Emb)*	*Arabidopsis thaliana*	Ath	Phytozome v12 (Goodstein et al., 2012)	27,416
	*Populus trichocarpa*	Ptr		41,335
	*Oryza sativa*	Osa		42,189
	*Sellaginella moellendorfii*	Smo		22,273
	*Physcomitrella patens*	Ppa		32,926
Chlorophyte (Chl)	*Volvox carteri*	Vca		14,247
	*Chlamydomonas reinhardtii*	Cre		17,741
Charophyte (Cha-KCM)	*Klebsormidium nitens* NIES 2285	Kni	*Klebsormidium nitens* transcripts V1.1 (Hori et al., 2014)	17,207
	*Mesostigma viride* NIES 995	Mvi^a^	SRR1594255 (Ju et al., 2015)	110,511
Charophyte (Cha-ZCC)	*Nitella mirabilis* S040	Nmi^a^	SRR486217, SRR494512 (Ju et al., 2015)	95,381
	*Coleochaete orbicularis* UTEX 2651	Cor^a^	SRR1594679 (Ju et al., 2015)	90,444
	*Spirogyra pratensis* UTEX 928	Spr^a^	SRR1594156 (Ju et al., 2015)	23,577
	*Z. circumcarinatum* SAG 2419	Zcir^a^	SRP117803 (Rippin et al., 2017)	67,762
	*Z. circumcarinatum* UTEX 1559	Zygy^a^	SRX5449751 (this study)	43,573

*^a^These six species have transcriptome (RNA-Seq) data and the other eight species have genomes.*

All-versus-all BLASTP analysis was performed for all proteins of the 14 species (*E*-value < 1e-5). All BLAST hits with an alignment coverage >50% with respect to the query were considered for further orthologous gene analysis. OrthoMCL was used to analyze the BLAST output and identify orthologous gene clusters (OGCs) (Li et al., 2003). According to which species the member proteins are from, OGCs were classified into nine groups:

(1) Chl+Cha+Emb clusters: with members found in chlorophytes (the Chlamydomonadales order) (Chl), charophytes (Cha), and embryophytes (Emb);
(2) Cha+Emb clusters: with members found in Cha and Emb, but not in Chl;
(3) Chl+Cha clusters: with members found in Chl and Cha, but not in Emb;
(4) Chl+Emb clusters: with members found in Chl and Emb, but not in Cha;
(5) Emb clusters: with members only found in Emb (≥2 species);
(6) Cha clusters: with members only found in Cha (at least one KCM species and one ZCC species);
(7) ZCC clusters: with members only found in ZCC clade of Cha (≥2 species) but not in KCM clade;
(8) KCM clusters: with members only found in KCM clade of Cha (≥2 species) but not in ZCC clade;
(9) Chl clusters: with members only found in Chl (≥2 species).

### Functional Annotation

We developed a workflow to annotate proteins of the above nine OGC groups for Gene Ontology (GO) functional descriptions (GO terms). For each OGC group, the DIAMOND program (Buchfink et al., 2014) was used to compare all the proteins to the UniProt database (Bateman et al., 2017). For each query protein, its UniProt hits that have the lowest *E*-values (*E*-value < 1e-10) and have associated GO terms were kept. The GO terms of these best UniProt hits were then assigned to the protein queries by parsing the UniProt ID mapping file downloaded from the UniProt database. Protein queries that did not have such UniProt hits were considered to be GO-unannotated and excluded from this analysis.

For GO enrichment analysis, the Chl+Cha+Emb OGCs were used as the control. For each of the other eight OGC groups, the R programming language function, “binom test” was used to compare the number of proteins with a specific GO term (limited to the 6th level of GO terms from biological process, cellular components, and molecular function categories) to the number of control group (i.e., Chl+Cha+Emb) proteins with the same GO term (R Core Team, 2018). The null hypothesis was that the tested GO term contains the same fraction of proteins in the tested OGC group and the control group. The “p.adjust” function in the R programming language was used to adjust for multiple comparisons (R Core Team, 2018). An adjusted *P*-value < 0.05 indicates an over-representation (or enrichment) of the tested GO term in the tested OGC group compared to the control group.

### Carbohydrate Active Enzyme (CAZyme) and Phylogenetic Analysis

Proteins of all 14 species were subjected to hmmscan against the dbCAN CAZyme HMM database (version 6.0, released 07/20/2017) (Yin et al., 2012). The output was filtered to select for domains with *E*-value < 1e-15 and alignment coverage >80% of the HMM domain. The resulted protein sequences of each of the three selected GT families were aligned with MAFFT v7.222 (Katoh and Standley, 2013). The FastTree program (Price et al., 2010) was used, with default parameters, to calculate approximately maximum-likelihood trees. FastTree performs Shimodaira-Hasegawa (SH) tests on each node to calculate SH-like local support values by 1,000 resamplings. The support values are shown in the phylogeny figures as fractions instead of percentages (e.g., 0.9 instead of 90%). The phylogenetic trees were uploaded and annotated using iTOL Web server version 3 (Letunic and Bork, 2016).

### Osmotic Stress Treatment and Differential Expression Measurement by qRT-PCR

Phylogenetic analysis of tree GT families were used for expression analysis and selected based on the representation of a subfamily. The draft nuclear genome of UTEX 1559 (unpublished data) was used as reference to assess exon-intron junctions via Exonerate software (model protein2genome; default settings) (Slater et al., 2005). The resulted gff files were filtered for exons exceeding 200 bp in length. Primer3 was used to design the qRT-PCR primers (parameters: product size min 60, opt 200, max 300; primer size min 18, opt 30, max 36; primer Tm min 77°C, opt 80°C, max 85°C; max self-complementarity 4.00). The sequences of the primers are listed in Supplementary Table S1. The qRT-PCR reactions were initially heated to 94°C for 2 min followed by 40 cycles of (94°C for 30 s, 55°C annealing temperature for 30 s, 72°C extensions for 60 s), using SYBR Green JumpStart Taq Readymix (Sigma) in a Mx3000P qRT-PCR system (Stratagene, Agilent Technologies, United States). Data were collected at the end of each annealing step. Primers for 18S rRNA were used as reference control. Genomic DNA and a water control were used to validate the primer specificity. Product sizes of qRT-PCRs were confirmed via agarose gels (1%, 125V for 30 min). Osmotic stress treatment was conducted by subjecting 3-week old *Z. circumcarinatum* cultures to 300 mM Sorbitol for 1 h and was regarded as short-term treatment. The cultures returned back to the growth chamber for 1 h prior to harvest. The control cultures were subjected to no treatment. Algae harvest and RNA extraction was performed in the same manner as described above. Data shown are the average of two technical replicates of three biological replicates with standard error (*n* = 6).

## Results

### Transcriptome Sequence Assembly

In total 55,575,710 reads were sequenced from the UTEX 1559 strain, which were assembled into 66,952 contigs/transcripts with an average contig length of 1,027 bp and N50 length of 2,011 bp (Table 1). The number of total assembled bases is 68,772,302 bp. Gene prediction in these contigs found 43,573 protein-coding genes, which may include falsely predicted ORFs from long non-coding RNAs. The same assembly and gene prediction pipeline was also used to analyze five other charophyte algal transcriptomes downloaded from NCBI and the numbers of predicted proteins are available in Table 2. These numbers are likely higher than the actual numbers of proteins encoded in the six charophyte species, given that the most recently published *C. braunii* genome was reported to contain fewer protein coding genes (23,546) (Nishiyama et al., 2018), and that the transcriptome data are often unable to reveal all the genes in the genomes.

### Orthologous Gene Clusters (OGCs) in Plants and Algae

In addition to the six charophyte (Cha) transcriptomes (one KCM species and five ZCC species), eight fully sequenced plant and algal genomes were also used in this study, which included the second KCM species (*K. nitens*), five embryophyte (Emb) species and two chlorophyte (Chl) species of the order Chlamydomonadales that served as the outgroup in this study. In total 646,562 predicted proteins of the 14 species were clustered based on sequence similarities for an orthologous gene cluster (OGC, equivalent to a protein family) analysis. As a result, 270,464 proteins were clustered into 25,485 OGCs (Table 3); each OGC contains at least two proteins from at least two species. The remaining proteins, which were either unclustered (i.e., singletons) or clustered with proteins from one single species, were excluded from the following analyses. These proteins have a higher chance of being falsely predicted from transcriptomes (e.g., ORFs from long non-coding RNAs) due to the lack of sequence homology in other species.

**Table 3:** Classification of orthologous gene clusters (OGCs) into nine groups.

OGC groups^a^	Max^#^ of species^b^	Total^#^ of proteins	Total^#^ of OGCs
Chl+Cha+Emb	14	115,796	5,031
Cha+Emb	12	66,491	4,174
Chl+Cha	9	10,323	1,221
Chl+Emb	7	1,030	140
Emb	5	28,954	4,849
Cha	7	22,077	3,600
Zcc	5	19,524	3,807
Kcm	2	257	66
Chl	2	6,012	2,597
Total		270,464	25,485

*^a^See “Materias and Methods” for the definition of the nine OGC groups. ^b^This shows the maximum number of species that an OGC can possibly have proteins from. For each OGC, the member proteins must be from ≥2 species but ≤ this number.*

**FIGURE 1:**
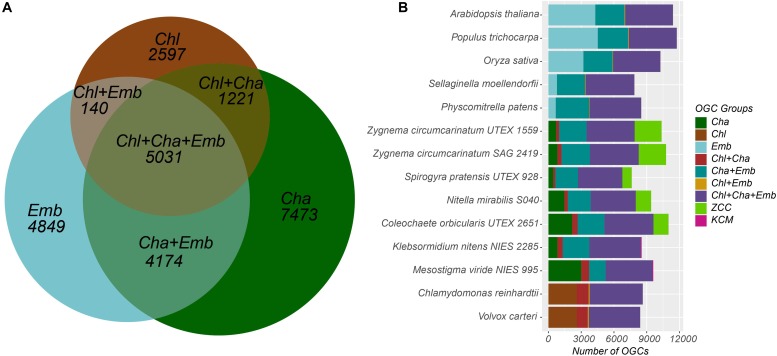
Distribution of the nine OGC groups in the 14 species. **(A)** The Venn diagram shows the numbers of OGCs shared by and unique to the three major plant/algal taxonomic groups. The 7473 OGCs unique to the Cha group are the sum of three OGC numbers (3600+3807+66) in Table 3. Note that the sizes of the seven areas in the diagram are proportional to the number of OGCs and not to the number of proteins. For example, the number of proteins in the Chl+Cha+Emb group is 115,769, which is the largest in Table 3. **(B)** For each species, the 25,485 OGCs were examined to see if there is a protein from that species. Then the OGC counts for that species were plotted with different OGC groups presented in different colors. The species labels in the y axis are arranged according to the phylogenetic relatedness. Note that the x-axis shows the number of OGCs not the number of proteins.

The 25,485 OGCs were further divided into nine groups according to what species the member proteins are from (see section “Materials and Methods” for details). For example, the Chl+Cha+Emb group contains 5,031 OGCs (Table 3 and Figure 1A), each of which must contain at least one species from the chlorophyte outgroup, one species from charophyte, and one species from embryophyte. Notably, OGCs of the Cha, ZCC, and KCM groups in Table 3 were combined as a single broader Cha group, which was labeled as Cha in the Venn diagram of Figure 1A. This broader Cha group has the largest number of OGCs (7,473 = 3,600 + 3,807 + 66 of Table 3), followed by the most conserved Chl+Cha+Emb group (5,031 OGCs), the Emb group (4,849 OGCs), and the Cha+Emb group (4,174 OGCs). This may simply reflect the fact that more species of charophytes and embryophytes were included in this analysis than the chlorophyte outgroup, and may also be due to the larger gene contents of charophytes and embryophytes (Table 2). The much smaller OGC numbers in the Chl+Cha group and Chl+Emb group (Table 3 and Figure 1A) agree with the idea that charophytes and embryophytes are closer sister groups than they are with the chlorophytes.

Since each OGC corresponds to a protein family, Figure 1B shows that the protein families shared by both taxonomic groups and the chlorophyte outgroup (i.e., the Chl+Cha+Emb OGC group in purple) are the most abundant in almost all species and exhibit a consistent trend throughout all the species included in this study. Again, there are more protein families shared between charophytes and embryophytes (i.e., the Cha+Emb group in blue-green) than those shared with chlorophytes (i.e., the Chl+Emb and Chl+Cha groups).

Unlike these more conserved families, the numbers of families unique in the different taxonomic groups varied significantly. For example, moss and spike moss have far fewer Emb-specific families (OGCs) than the three, later evolved plants (Arabidopsis, rice, and poplar). In charophytes, we have separated the broader Cha group into Cha, ZCC, and KCM groups in Figure 1B. The counts of the Cha specific families (dark-green) fluctuated quite remarkably from species to species. Notably, *Mesostigma viride* shares very few protein families with the other KCM species *K. nitens* (the KCM group in magenta), but shares a large number of families with ZCC species (the Cha group in dark-green). Additionally, the ZCC specific families (light-green) are more abundant in ZCC than the KCM specific families (magenta) in KCM species, which may be a sampling artifact, as more ZCC species were included in this study.

### Cell Wall-Related Gene Ontology (GO) Functions Are Highly Enriched in Protein Families Shared by Charophytes and Embryophytes (i.e., the Cha+Emb Group of OGCs)

To understand the functional differences among the nine groups of OGCs, we have performed GO annotation enrichment analysis for proteins in the OGCs. UniProt was used as the database in the sequence similarity search, which was the first step for the GO annotation; then GO terms of the UniProt protein hits were transferred to the OGC protein queries (see section “Materials and Methods” for details). As the UniProt database contains a large number of proteins from sequenced embryophyte and chlorophyte genomes, OGCs from Emb, Chl, and Chl+Emb groups have much higher percentages of GO-annotated proteins (all higher than 73%, Table 4). In contrast, these percentages are much lower for OGC groups containing charophytes, which have very few sequences available in UniProt.

**Table 4:** Overview of gene ontology (GO) annotation for OGCs of the nine groups.

OGC groups	^#^Of GO annotated proteins	% Of GO annotated proteins	^#^Of over-represented GO terms^b^
Chl+Cha+Emb	80,871	69.84%	NA^a^
Cha+Emb	41,532	62.46%	501
Chl+Cha	4,248	41.15%	76
Chl+Emb	907	88.06%	65
Emb	24,831	85.76%	404
Cha	3,785	17.14%	20
ZCC	6,201	31.76%	42
KCM	85	33.07%	6
Chl	4,440	73.85%	54

*^a^It is not available because the Chl+Cha+Emb OGC group is used as the control (to be compared with) for the other eight OGC groups in the GO term enrichment analyses (see section “Materials and Methods” for details). ^b^Adjusted P-value < 0.05.*

The major goal of the OGC and GO enrichment analyses was to find what functional differences exist among different OGC groups, which, in turn, can shed light on the gene content innovations occurred during algal and land plant evolution. To this end, we have used the most conserved Chl+Cha+Emb group as the control and compared all the other eight groups against it to identify GO terms that are significantly enriched/over-represented (the last column of Table 4 and Supplementary Data Sheet S1). Particularly, we were interested in GO terms over-represented in the Cha+Emb group of OGCs, which correspond to protein families emerged in the common ancestor of charophytes and embryophytes (because they are absent in the chlorophyte outgroup).

**FIGURE 2:**
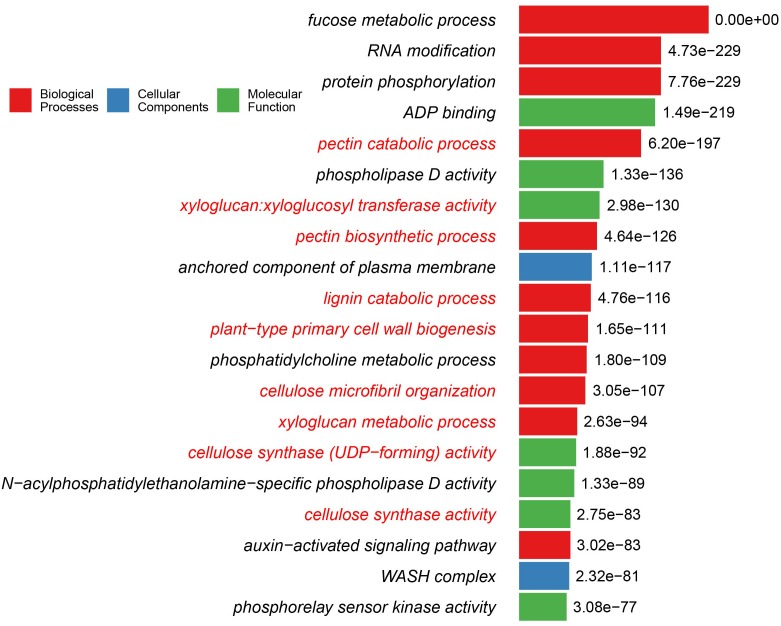
Top 20 GO functions/terms that are over-represented in the Cha+Emb group of OGCs. The y-axis shows the GO terms, and the binomial test adjusted *P*-values in -log10 form are shown beside the bars. The bars are color-coded according to which of the three top GO levels the term is from. The GO terms are highlighted in red fonts if they are cell wall-related functions. The detailed methods for this GO enrichment analysis is described in Methods. Detailed data used for this plot is available in Supplementary Data Sheet S1.

It is intriguing to note that plant cell wall-related GO terms are highly over-represented in the Cha+Emb group of OGCs (Supplementary Data Sheet S1). Nine of the top 20 GO terms of the Cha+Emb group are related to cellulose, pectin, xyloglucan, and lignin metabolisms (Figure 2). As a comparison, in the Cha and ZCC group top 20 lists, only one GO term (cellulose microfibril organization) is significantly enriched, and in the Emb group top 20 list, three GO terms related to the pectin and xyloglucan metabolisms are enriched (Supplementary Data Sheet S1), and in the other groups there are none.

Interestingly, some GO terms in Figure 2 such as cellulose synthase activity, plant-type primary cell wall biogenesis, pectin biosynthetic process, cellulose synthase (UDP-forming) activity are also significantly enriched in the Emb group but to a lesser extent (i.e., with less significant *P*-values and not present in the top 20 list). The lignin catabolic process and the cellulose microfibril organization GO terms of Figure 2 are not over-represented in the Emb group.

When looking at all GO terms with significant *P*-values (Supplementary Data Sheet S1), there are a total of 32 cell wall-related GO terms in the Cha+Emb group, which include: biosynthetic, catabolic, and regulatory processes for most of the major cell wall polymers such as cellulose, pectin, lignin, xyloglucan, and xylan. Similarly, there are 27 cell wall-related GO terms over-represented in the Emb group, which also involve mannan in addition to the above cell wall polymers. Interestingly, in comparison to the Emb group, the Cha+Emb group has more enriched GO terms that are related to cellulose (5 terms in Cha+Emb vs. 2 terms in Emb), pectin (5 vs. 4), xyloglucan (3 vs. 2), and lignin (2 vs. 1). On the other hand, in comparison to the Cha+Emb group, the Emb group has more enriched GO terms that are related to xylan (4 vs. 3), mannan (2. vs. 0), and secondary cell wall (3 vs. 0).

### Expression of Cell Wall Polysaccharide Biosynthesis-Related GT Enzymes Is Upregulated During Osmotic Stress in UTEX 1559

The above GO functional enrichment analysis found that plant cell wall-related gene families are highly enriched in the Cha+Emb group and thus interpreted as new inventions/additions evolved in charophytes. As an experimental validation of their expression in UTEX 1559, we selected 15 genes (Table 5) from three GT protein families (GT2, GT8, and GT43) that contain the most important enzymes for cell wall polysaccharide synthesis (see section “Introduction”) and conducted a differential gene expression study using qRT-PCR analysis. Since cell walls are critical to protect plant cells against osmotic stress, we intended to study the expression of these 15 cell wall genes in UTEX 1559 with Sorbitol treatment compared with the control (no Sorbitol treatment).

**Table 5:** Selected GT enzyme encoding genes from UTEX 1559 and their best Arabidopsis homologs.

GT family	Gene name	Length (in aa)	Best hit in Arabidopsis	Arabidopsis protein length	Sequence identity (%)	BLASTP E-value
GT2	ZcCesA	1137	AT5G05170 (AtCesA3)	1065	63	0.0
	ZcCesA-like	280	AT4G32410 (AtCesA1)	1081	52	7e-97
	ZcCslC	715	AT2G24630 (AtCslC8)	690	61	0.0
	ZcCslA-like	666	AT2G35650 (AtCslA7)	484	45	2e-106
GT8	ZcGAUT3	731	AT4G38270 (AtGAUT3)	676	59	0.0
	ZcGAUT10	624	AT2G20810 (AtGAUT10)	536	50	2e-178
	ZcGAUT13	562	AT3G01040 (AtGAUT13)	532	50	0.0
	ZcGATL7-like	423	AT3G62660 (AtGATL7)	361	27	7e-06
	ZcGolS	345	AT1G56600 (AtGolS2)	335	46	8e-84
	ZcPGSIP-A-like	924	AT1G08990 (AtPGSIP5)	566	35	2e-37
	ZcPGSIP-B	555	AT5G18480 (AtPGSIP6)	537	49	5e-159
	ZcPGSIP-C	510	AT4G16600 (AtPGSIP8)	494	47	4e-154
GT43	ZcGT43-A	501	AT1G27600 (AtRX9L)	394	65	1e-124
	ZcGT43-B	711	AT5G67230 (AtIRX14L)	492	35	2e-82
	ZcGT43-C	485	AT1G27600 (IRX-9)	394	32	1e-35

**FIGURE 3:**
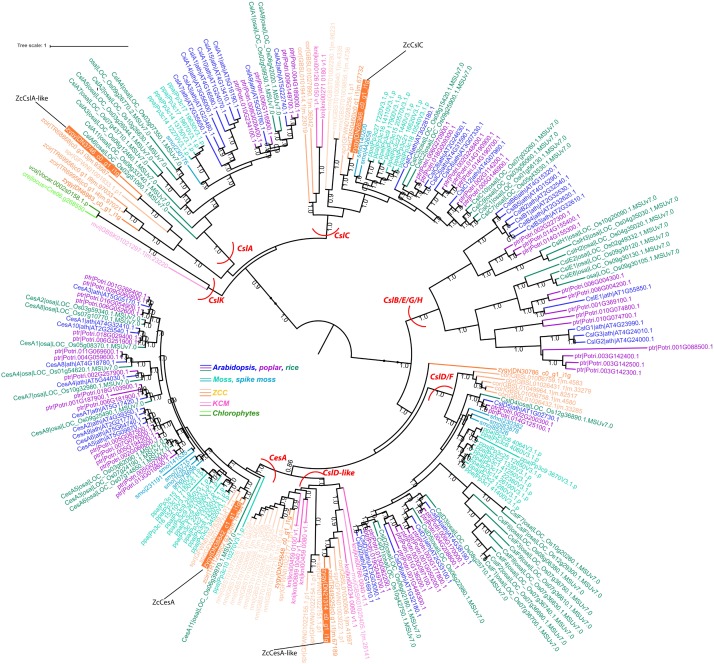
The phylogeny of GT2 proteins from selected species of land plants and algae. In total 248 GT2 protein sequences of 14 plant and algal species were used to build this phylogeny (see section “Materials and Methods”). For Arabidopsis and rice proteins, the gene names (adopted from Wang et al., 2010; Yin et al., 2014) were included in the tree leaves. The UTEX 1559 proteins that were selected for qRT-PCR analysis were highlighted with orange background and the proposed gene names (Table 5) were indicated with black lines.

The 15 genes were selected based on strict phylogenetic analyses to cover UTEX 1559 orthologs of as many GT subfamilies as possible, which have been continuously determined by our research group since 2009 (Yin et al., 2009, 2010, 2014; Taujale and Yin, 2015). For example, as shown in Figure 3, the GT2 phylogeny contains seven UTEX 1559 proteins of GT2. We selected four of them representing CesA, CslD-like, CslC, and CslA subfamilies (Table 5), which were defined in our earlier paper (Yin et al., 2014). The orthologs (ZcCesA and ZcCslC) for CesA and CslC subfamilies were evident as the phylogenetic clusterings were strongly supported (SH test support values = 1.0, see section “Materials and Methods”). Between the CesA and CslD/F clades, we observed a charophyte-specific cluster, which was named CslD-like clade in our previous paper, as it was clustered with CslD/F clade, but with a low support value (Yin et al., 2014). In this study, the CslD-like clade is clustered with the CesA clade with a robust support (0.86), and the selected protein of this CslD-like clade is more similar to AtCesA proteins than to AtCslD proteins and thus named ZcCesA-like (Table 5). Additionally, in 2014, we did not find CslA orthologs in charophytes. By including more charophyte transcriptome data in this study, we have now identified a cluster of Zygnematophyceae proteins sister to the land plant CslA clade, which has very low support (0.10) though. Notably, this calde is also distinct from the chlorophyte-specific CslK clade (Yin et al., 2014). Therefore, we are still unsure of the existence of CslA orthologs in charophytes. Nevertheless, the selected protein has its best Arabidopsis homolog to be AtCslA7 (Table 5) and thus is named ZcCslA-like.

Using the same idea, eight (out of 12) GT8 genes (Supplementary Figure S1) and three (out of ten) GT43 genes (Supplementary Figure S2) were also selected (Table 5). For GT8 genes, the seven major clades were adopted from our previous paper (Yin et al., 2010). The GAUT clade was further grouped into subclades, and the ZcGAUT3, ZcGAUT10, and ZcGAUT13 genes were named after their best Arabidopsis homologs (Table 5). These three ZcGAUT genes and ZcGolS, ZcPGSIP-B, ZcPGSIP-C are all unambiguous orthologs of their respective clades/subclades because the phylogenetic clusterings are all supported with robust supporting values. However, ZcGATL7-like (Arabidopsis homolog AtGATL7) is not clustered with the land plant GATL clade. Instead, it is phylogenetically more related to the GATR (GAUT and GATL-related) clade. Similarly, ZcPGSIP-A-like is not clustered with the land plant PGSIP-A clade, although its closest Arabidopsis homolog is AtPGSIP5 of PGSIP-A.

For GT43 genes, we followed the nomenclature in our previous paper (Taujale and Yin, 2015), where we defined three major GT43 clades. All the three selected genes, ZcGT43-A (ortholog of AtIRX9/9L), ZcGT43-B (ortholog of AtIRX14/14L), and ZcGT43-C (charophyte-specific clade), are clustered within their corresponding clades with robust support values.

**FIGURE 4:**
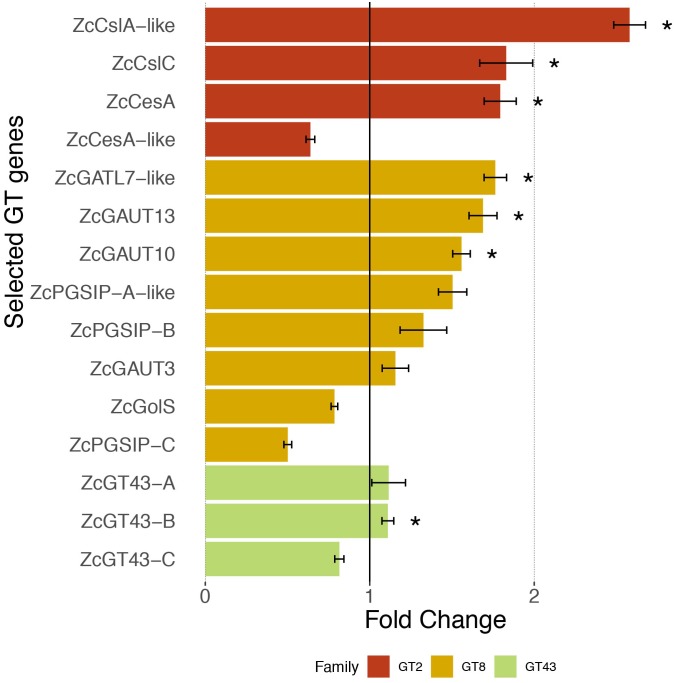
Expression response of 15 selected GT genes toward osmotic stress. Quantitative RT-PCR measures the relative expression in fold change (average ± SE, *n* = 6) in UTEX 1559 after 1 h of 300 mM Sorbitol treatment vs. no treatment (control). Significant *p*-values (<0.05) from *t*-tests were shown as ^∗^ to indicate that the Sorbitol treatment samples were significantly higher than control samples in terms of expression.

Figure 4 shows that three out of the four selected ZcGT2 genes had over 1.5 fold expression increase (with significant *P*-values) after 1 h Sorbitol treatment, of which the ZcCslA-like (land plant CslA encodes mannan synthase) showed the highest (more than 2.5 times higher) fold change in response to the osmotic stress. ZcCesA (cellulose synthase) and ZcCslC (xyloglucan synthase) also had a near two-fold expression increase. However, the fourth gene ZcCesA-like showed a down-regulation after 1 hr stress treatment. We noted that the ZcCesA-like protein is only 280 amino acids in length (Table 5), which is likely a partially assembled transcript fragment; the PCR primer designed for this gene may not have provided a complete sequence when compared to other assembled transcripts having greater lengths.

For the eight ZcGT8 genes related to pectin and xylan synthesis, after Sorbitol treatment, four of them (ZcGATL7-like, ZcPGSIP-A-like, ZcGAUT10, ZcGAUT13) showed over 1.5 fold up-regulation (three have significant *P*-values), whereas two (ZcGolS, ZcPGSIP-C) showed down-regulation. Overall the degree of ZcGT8 up-regulation is lower than that of ZcGT2. Lastly, none of the three ZcGT43 genes (related to xylan synthesis) showed more than 1.5 fold up-regulation, although one of them (ZcGT43-B) has a significant *P*-value.

## Discussion

Charophyte green algae (CGA) are sister clade to all land plants. Thus, genome and transcriptome data of charophytes are critical to understanding how land plants have evolved. The goal of this study was to sequence the transcriptome of *Z. circumcarinatum* UTEX 1559 and use it as a comparison across genome/transcriptome data of other charophytes, land plants and a chlorophyte outgroup, to identify functions that are shared by land plants and CGA with a special focus on cell wall biosynthesis-related GT enzymes.

### Cell Wall-Related GO Functions Are Among the Top Protein Families That Are Shared by Land Plants and Charophytes

By clustering the protein sequences of selected 14 plant and algal species into orthologous gene clusters (OGCs), we were able to classify 25,485 OGCs into nine groups. We paid particular attention to OGCs containing at least one embryophyte species and one charophyte species, i.e., the Cha+Emb group, which contains OGCs that evolved in charophytes and remained in embryophytes. By comparing the GO annotation of OGCs in the Cha+Emb group and the Chl+Cha+Emb group (OGCs present in embryophytes, charophytes, and chlorophyte outgroup), we found 501 GO terms significantly over-represented in the Cha+Emb group, which correspond to gene families evolved or expanded in charophytes. Very interestingly, we found 32 cell wall-related GO terms highly enriched in the Cha+Emb group. These terms describe biological processes for the biosynthesis of most of the major plant cell wall polymers including cellulose, pectin, lignin, xylan, and xyloglucan. This supports and extends previous biochemical and genetic analysis results, showing that the molecular machineries for the synthesis of cellulose, hemicellulose, pectin, and lignin have already evolved in charophytes (Sørensen et al., 2011; Mikkelsen et al., 2014).

For example, lignin-like compounds have been found in different species of *Coleochaete* and *Nitella* decades ago (Delwiche et al., 1989; Ligrone et al., 2008). More recently this was confirmed in more *Coleochaete* species using immunofluorescence of an anti-lignin agent as well as a thioacidolysis method that allows for the detection of guaiacyl (G) and syringyl (S) lignin monomers (Sørensen et al., 2011). Additionally, a recent charophyte transcriptome data mining and phylogenetic analysis has suggested that key genes in the lignin biosynthetic pathway have already evolved in charophytes (De Vries et al., 2017).

Another example is xylan, which is the second most abundant polysaccharides found in land plant cell walls. Xylans have been suggested to exist in *Spirogyra sp.* and some other charophyte species using glycan microarray assay with cell walls extracted using cadoxen (Sørensen et al., 2011). At least four different GT8 families (GT47, GT43, GT8, GT61) are involved in xylan biosynthesis (Jensen et al., 2018). Our phylogenies of GT8 (Supplementary Figure S1), GT43 (Supplementary Figure S2), and GT47 (Supplementary Figure S3) indicated that *Zygnema* and *Spirogyra* have unambiguous orthologs of AtIRX8/GAUT12 (GT8), AtIRX9 (GT43), AtGUT2/AtIRX10/AtXYS1 (GT47), and AtIRX14/14L (GT43), which might be related to xylan backbone synthesis. It was interesting to note that the sequenced *K. nitens* genome also has orthologs for these four putative backbone synthases as well as AtIRX7/AtFRA8 (GT47), while the sequenced *C. braunii* genome does not have orthologs of any of these genes. The side chain of xylans differs significantly between dicot and monocot plants. AtGUX1-5 (GT8) only have *Spirogyra* orthologs (Supplementary Figure S1), whearus grass XAT and XAX proteins (GT61) do not have any charophyte orthologs (Supplementary Figure S4), suggesting the xylan side chain biosynthetic enzymes might have evolved much later than enzymes for backbone synthesis.

### Only Zygnematophyceae Has Land Plant CesA Orthologs and Other CGA CesAs Form a CGA-Specific Csl Subfamily

**FIGURE 5:**
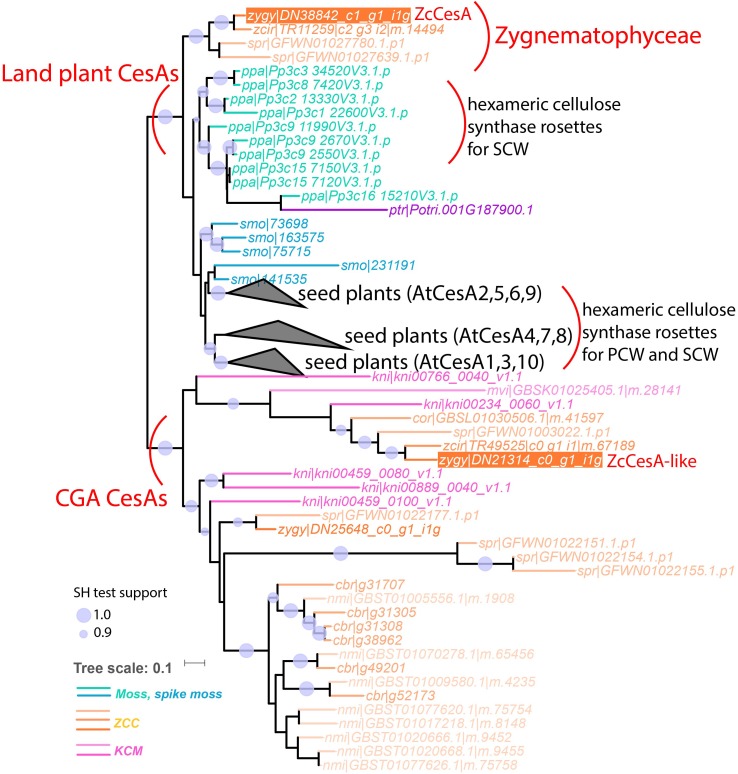
The phylogeny with land plant CesAs and CGA CesAs (former CslD-like clade). In total 85 protein sequences of 15 plant and algal species were used to build this phylogeny (see section “Materials and Methods”). These include six proteins (leaf names contain “cbr| ”) from the sequenced *C. braunii* genome and 79 proteins from the CesA and CslD-like clades of Figure 3. The three seed plant clades are collapsed as black triangles with the representative Arabidopsis proteins indicated (AtCesA 4, 7, 8 are reportedly involved in secondary cell wall cellulose synthase complex (CSC) assembly, and the rest AtCesAs are involved in primary cell wall CSC assembly). The complete version of this phylogeny is Supplementary Figure S5.

Further detailed phylogenetic analyses of GT2, GT8, and GT43 protein families proved that UTEX 1559 has orthologs in all the three families. Within GT2, UTEX 1559 has a CesA ortholog, together with two other Zygnematophyceae (*Z. circumcarinatum* SAG 2419 and *S. pratensis* UTEX 928), clustered with land plant CesAs with strong support (Figure 3). Our previous analysis showed that *Penium margaritaceum* of Zygnematophyceae was also found in this cluster (Yin et al., 2014). Interestingly, in this land plant CesA cluster there are no other charophyte species, not even the *K. nitens* NIES-2285 that has the sequenced genome. Furthermore, adding GT2 homologs of the recent *C. braunii* genome in the phylogeny did not reveal any orthologs in this cluster either (Figure 5). Therefore, all these suggest that the land plant CesA orthologs only exist in Zygnematophyceae, but not in Coleochaetophyceae and Charophyceae of the ZCC clade, nor in the KCM clade. This raised an interesting question: where are the CesAs in the remaining charophytes? We believe that the previously defined CslD-like clade is the answer (Yin et al., 2014). Firstly, it is a charophyte-specific clade and has all the charophyte species that were included in this analysis (Figure 3, 5). Secondly, it is phylogenetically clustered with the land plant CesA clade in this study (Figure 3), although in our previous paper it is clustered with land plant CslD clade but with a weak support. It is highly possible that this CslD-like clade (renamed as CGA CesA clade in Figure 5) actually represents a charophyte-specific CesA clade, which have separated from the land plant CesA family (have Zygnematophyceae orthologs) and the land plant CslD family (have Coleochaetophyceae orthologs according to Figure 3) prior to the emergence of land plants.

One potential evolutionary model could explain the presence of these CesAs as having evolved in an ancestral CGA line, which were all derived from the CGA CesA clade (Figure 5) through divergent evolution. One of the branches in the CesA clade was only found in ancestral Zygnematophyceae and eventually became the ancestor of all land plant CesAs. Another branch became the ancestor of all land plant CslDs (modern Coleochaetophyceae still has CslD but *C. braunii* of Charophyceae does not). Although the molecular function of land plant CslD is unknown, CslD is phylogenetically closest to CesA, has the closest sequence length to CesA, has orthologs (at least) in Coleochaetophyceae, and indirect evidence has suggested that CslD may be responsible for non-crystalline cellulose synthesis (Manfield et al., 2004; Yagisawa et al., 2009) or mannan synthesis (Verhertbruggen et al., 2011; Yin et al., 2011; Li et al., 2017).

Additionally, although the two recent genome papers (Bowman et al., 2017; Nishiyama et al., 2018) indicated the emergence of cellulose synthase rosettes prior to the ZCC clade, we believe there is no direct evidence for this. Even if there are cellulose synthase rosettes in ZCC, the land plant-like CesA only exist in Zygnematophyceae (Figure 5), and it is unknown if the Zygnematophyceae CesAs form cellulose synthase complex (CSC) in a similar fashion to seed plants, and if the CSC synthesizes microfibril cellulose. In this regard, it is interesting to note that the six-lobed rosette structure has been observed in moss *P. patens* (Nixon et al., 2016), although the moss CSC subunits do not phylogenetically correspond to seed plant CSC subunits, suggesting the convergent evolution of cellulose synthase rosettes in different taxonomic groups (Norris et al., 2017).

### Enriched GO Terms Also Include Function Related to Plant Hormones, Desiccation/Drought, Biotic and Abiotic Stresses, and Filamentous Actin (for Forming Preprophase Band and Phragmoplast in Cell Division)

Although cell wall-related GT enzymes are the focus of this study, the GO enrichment analysis also revealed other important functions having over-representation in the Cha+Emb group. In Figure 2, the auxin-activated signaling pathway is significantly enriched in the Cha+Emb group (adjusted *P*-value = 3.02E-83), so are the phospholipase D activity (1.33E-136) and the phosphatidylcholine metabolic process (1.80E-109), which are related to cold and salt stress (Munnik et al., 2000; Meijer and Munnik, 2003; Guo and Wang, 2012; Muzi et al., 2016; Ben Othman et al., 2017). In fact, looking down the list in Supplementary Data Sheet S1 (colored in red background), we observed signaling, biosynthesis, transporting, and regulating pathways for more plant hormones (such as ethylene, cytokinin, jasmonic acid, gibberellic acid, brassinosteroid, abscisic acid, indoleacetic acid) in the Cha+Emb and Emb groups. This agrees with previous papers that many genes of major plant hormone related pathways have already existed in charophytes (Hori et al., 2014; Ju et al., 2015), although, compared to Cha+Emb group, the Emb group has even more significantly enriched GO terms related to plant hormones. It underlies the importance of plant hormones in response to various abiotic stresses such as desiccation and osmotic stress (Verma et al., 2016; Bielach et al., 2017), which must have been essential for the ancestral charophytes, that gave rise to land plants, to transition and adapt to harsh terrestrial environments.

Indeed, we have found GO term enrichment toward various abiotic stresses such as heat, cold, light, desiccation, drought, water deprivation, salt, oxidative and osmotic stresses in the Cha+Emb group (Supplementary Data Sheet S1, colored in orange and green background). The desiccation enrichment is in line with the notion that the ancestral charophytes had occupied mostly outer rims of freshwater habitats where they had to cope with prolonged periods of drought (Pichrtová et al., 2014; Delwiche and Cooper, 2015). This intermittently wet lifestyle must have demanded the development of drought tolerance and recovery machinery to persist dry periods (Delwiche and Cooper, 2015). The enrichment of cold, light, and desiccation functions agrees with the previous differential expression studies made in two *Z. circumcarinatum* strains (Rippin et al., 2017; de Vries et al., 2018).

Interestingly, we also observed the enrichment of GO terms in regard to biotic stress, such as response to fungi, oomycetes, and viruses in the Cha+Emb group (Supplementary Data Sheet S1, colored in blue background). As expected, there are more enriched GO terms on biotic stress in the Emb group. This indicates that the recognition of and potential interaction with microbes have already evolved in charophytes. Interestingly, it has been shown that fungal hyphae are present in *Nitella tenuissima* (Knack et al., 2015). Furthermore, orthologs of signaling modules of nuclear envelope-localized potassium channel (DMI1) and calcium- and calmodulin CCaMK have been found in *Spirogyra* sp., marking the important evolutionary step toward symbiotic relationship with beneficial symbionts (Delaux et al., 2015).

The recent Chara genome paper observed the actin protein family expansion, which was explained to enhance cytoplasmic streaming (Nishiyama et al., 2018). In addition, ZCC and land plants were grouped together as the Phragmoplastophyta due to the observation of phragmoplasts and preprophase band (PPB) of microtubules in at least some ZCC algae (Buschmann and Zachgo, 2016). Interestingly, in the Cha+Emb group our GO enrichment analysis found eight significantly enriched GO terms that are phragmoplasts and PBB related [e.g., WASH complex (2.32E-81) and F-actin capping protein complex (4.44E-14), Supplementary Data Sheet S1, colored in red font]. Three of these GO terms are also enriched in the ZCC group. Therefore, our finding provides further and robust support for the emergence of phragmoplasts and PBB of microtubules in ZCC. This is also consistent with the enrichment of cellulose synthesis-related functions and abiotic stress-related functions, because actins and cortical microtubules coordinate the delivery of CesA complexes to the plasma membrane and affect the arrangement of cell wall polysaccharides in response to extracellular environmental change (e.g., osmotic stress) (Gutierrez et al., 2009).

## Data Availability

The datasets generated for this study can be found in NCBI SRA, SRX5449751.

## Author Contributions

EF conceived and conducted the RNA-Seq and qRT-PCR experiments, data analysis and wrote the manuscript. LO, SE, and CA contributed to the data analysis. WG contributed to the RNA-Seq and qRT-PCR experiments. MD advised LO and contributed to the manuscript writing. YY secured the grant, conceived the study, participated in the data analysis, advised EF, LO, SE, CA, and wrote the manuscript. All authors read and approved the manuscript.

## Conflict of Interest Statement

The authors declare that the research was conducted in the absence of any commercial or financial relationships that could be construed as a potential conflict of interest.

## Footnotes

1 www.arabidopsis.org

## Supplementary Material

FIGURE S1 The phylogeny of GT8 proteins from selected species of land plants and algae. In total 262 GT43 protein sequences of 14 plant and algal species were used to build this phylogeny (see section “Materials and Methods”). For Arabidopsis and rice proteins, the gene names (adopted from Yin et al., 2010) were included in the tree leaves. Arabidopsis proteins that are known to be xylan biosynthesis-related were indicated in red (AtIRX8, AtGUX1-5, and AtPARVUS). The UTEX 1559 proteins that were selected for qRT-PCR analysis were highlighted with orange background and the proposed gene names (Table 5) were indicated with black lines.

FIGURE S2 The phylogeny of GT43 proteins from selected species of land plants and algae. In total 52 GT43 protein sequences of 14 plant and algal species were used to build this phylogeny (see section “Materials and Methods”). For Arabidopsis proteins, the gene names (adopted from Taujale and Yin, 2015) were included in the tree leaves. The UTEX 1559 proteins that were selected for qRT-PCR analysis were highlighted with orange background and the proposed gene names (Table 5) were indicated with black lines.

FIGURE S3 The phylogeny of GT47 proteins from selected species of land plants and algae. In total 522 GT47 protein sequences of 14 plant and algal species were used to build this phylogeny (see section “Materials and Methods”). For Arabidopsis proteins, the gene names (adopted from Møller et al., 2017) were included in the tree leaves. Arabidopsis proteins that are known to be xylan biosynthesis-related were indicated in red (AtIRX7, AtIRX10, and AtIRX10-like).

FIGURE S4 The phylogeny of GT61 proteins from selected species of land plants and algae. In total 74 GT61 protein sequences of 14 plant and algal species were used to build this phylogeny (see section “Materials and Methods”). The A and C clades classification was adopted from Chiniquy et al. (2012). The characterized grass XAT and XAX were indicated to be present in the clade C.

FIGURE S5 The phylogeny with land plant CesAs and CGA CesAs (former CslD-like clade). This is the complete version of Figure 5.

TABLE S1 qRT-PCR primers of the 15 GT candidate genes.

DATA SHEET S1 Significantly enriched GO terms in the eight OGC groups vs. the control (Chl+Cha+Emb). There are 10 sheets, including eight sheets for the eight OGC group, and two additional sheets with selected GO terms from the Cha+Emb group and the Emb group. The selected GO functions are highlighted with different colors and discussed in the paper.
