# Supplementary figures and images for: Cell Wall Enzymes in Zygnema circumcarinatum UTEX 1559 Respond to Osmotic Stress in a Plant-Like Fashion
Source: Front Plant Sci. 2019 Jun 7;10:732. doi: 10.3389/fpls.2019.00732 (PMC6566377; doi:10.3389/fpls.2019.00732)

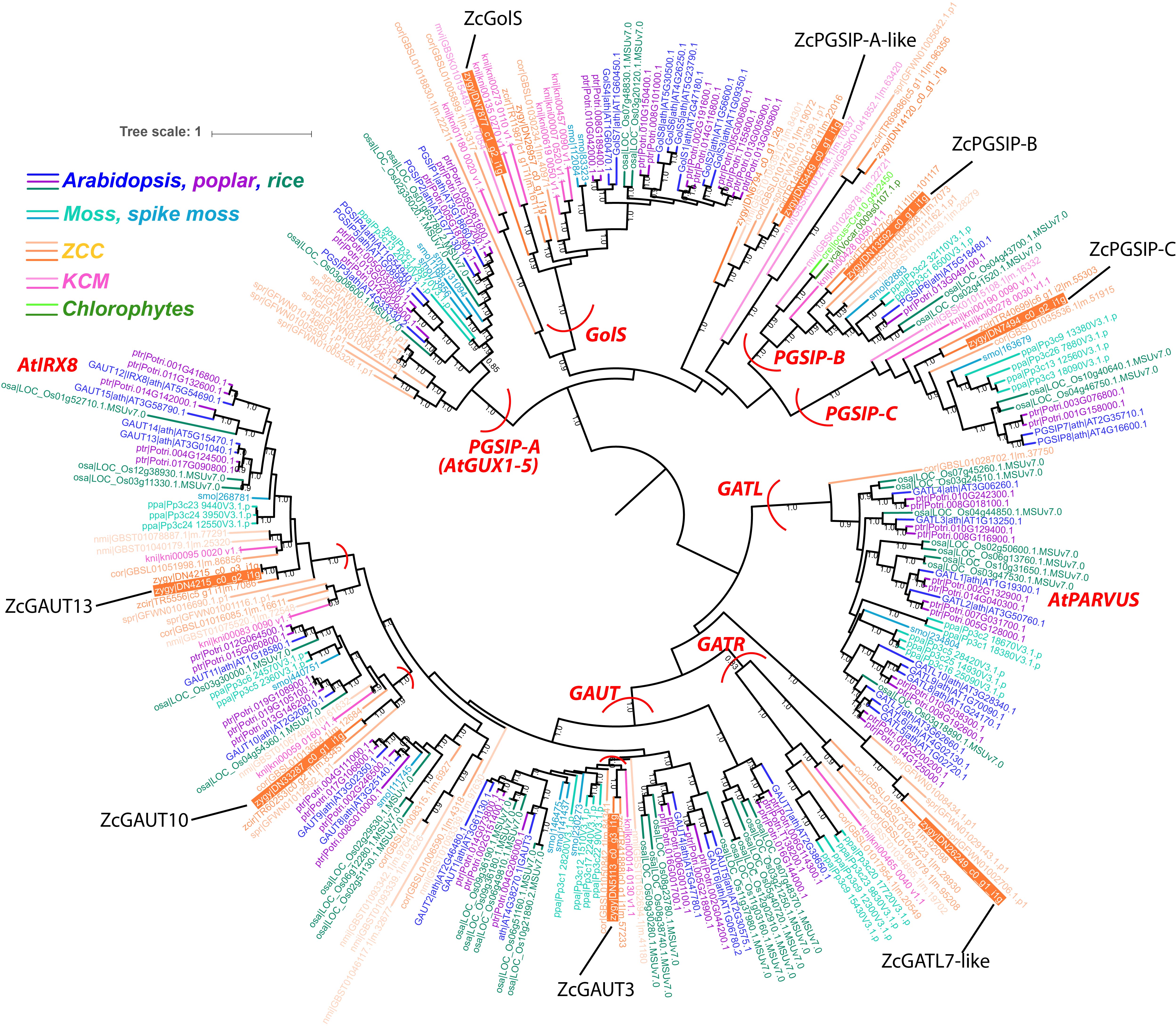

Supplement: FIGURE S1 — The phylogeny of GT8 proteins from selected species of land plants and algae. In total 262 GT43 protein sequences of 14 plant and algal species were used to build this phylogeny (see section “Materials and Methods”). For Arabidopsis and rice proteins, the gene names (adopted from Yin et al., 2010) were included in the tree leaves. Arabidopsis proteins that are known to be xylan biosynthesis-related were indicated in red (AtIRX8, AtGUX1-5, and AtPARVUS). The UTEX 1559 proteins that were selected for qRT-PCR analysis were highlighted with orange background and the proposed gene names (Table 5) were indicated with black lines. [file Image_1.JPEG]

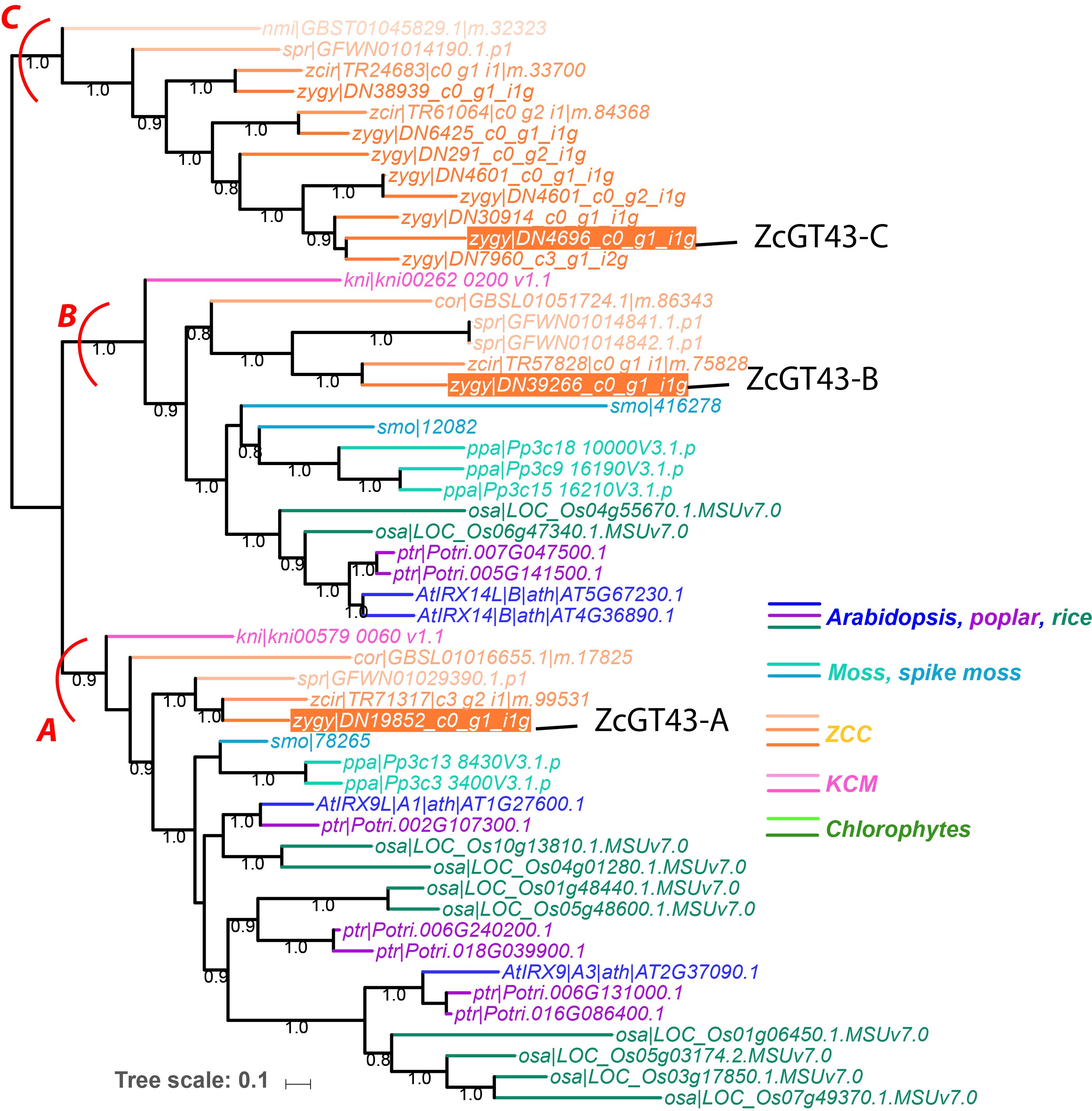

Supplement: FIGURE S2 — The phylogeny of GT43 proteins from selected species of land plants and algae. In total 52 GT43 protein sequences of 14 plant and algal species were used to build this phylogeny (see section “Materials and Methods”). For Arabidopsis proteins, the gene names (adopted from Taujale and Yin, 2015) were included in the tree leaves. The UTEX 1559 proteins that were selected for qRT-PCR analysis were highlighted with orange background and the proposed gene names (Table 5) were indicated with black lines. [file Image_2.JPEG]

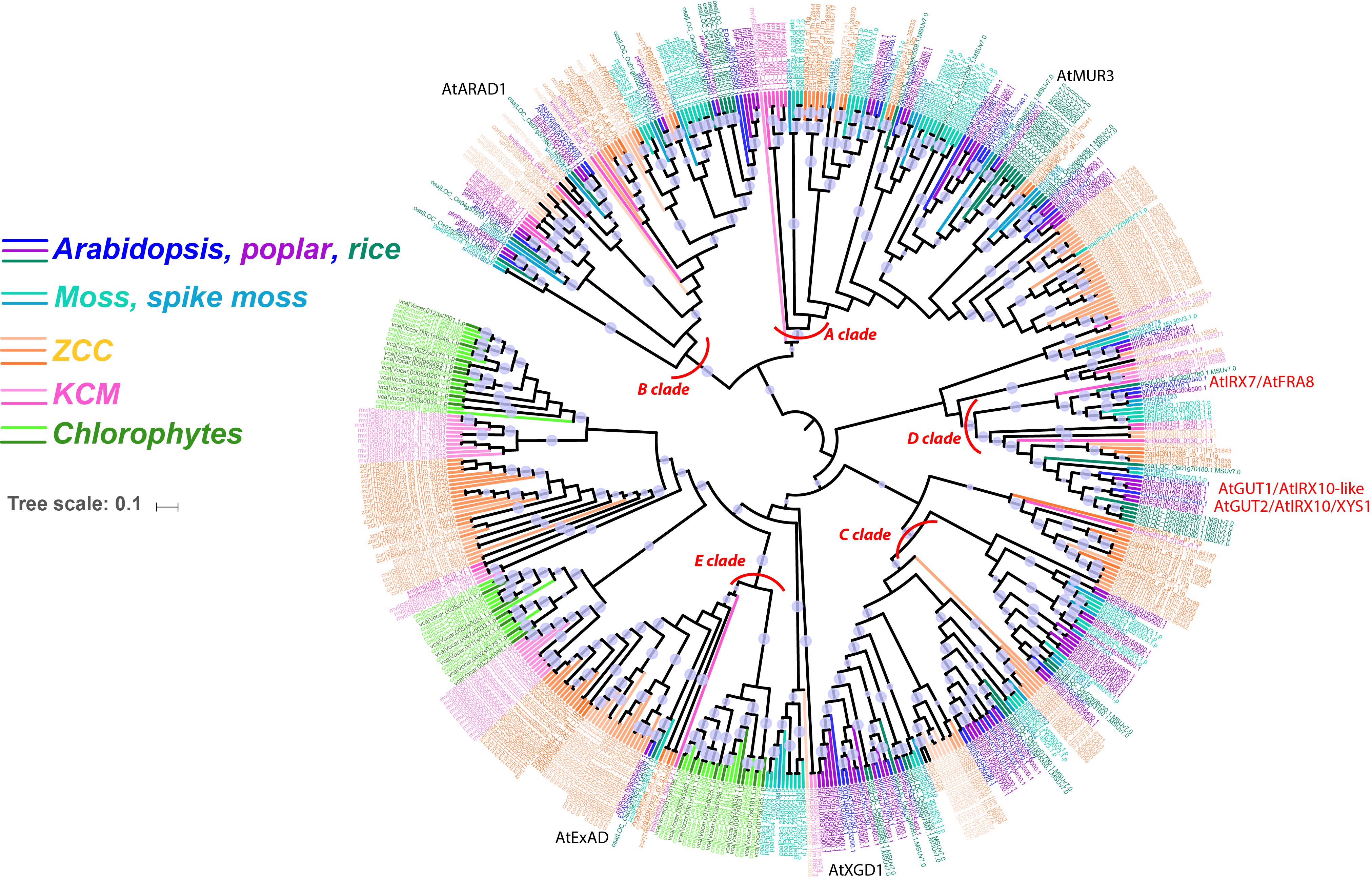

Supplement: FIGURE S3 — The phylogeny of GT47 proteins from selected species of land plants and algae. In total 522 GT47 protein sequences of 14 plant and algal species were used to build this phylogeny (see section “Materials and Methods”). For Arabidopsis proteins, the gene names (adopted from Møller et al., 2017) were included in the tree leaves. Arabidopsis proteins that are known to be xylan biosynthesis-related were indicated in red (AtIRX7, AtIRX10, and AtIRX10-like). [file Image_3.JPEG]

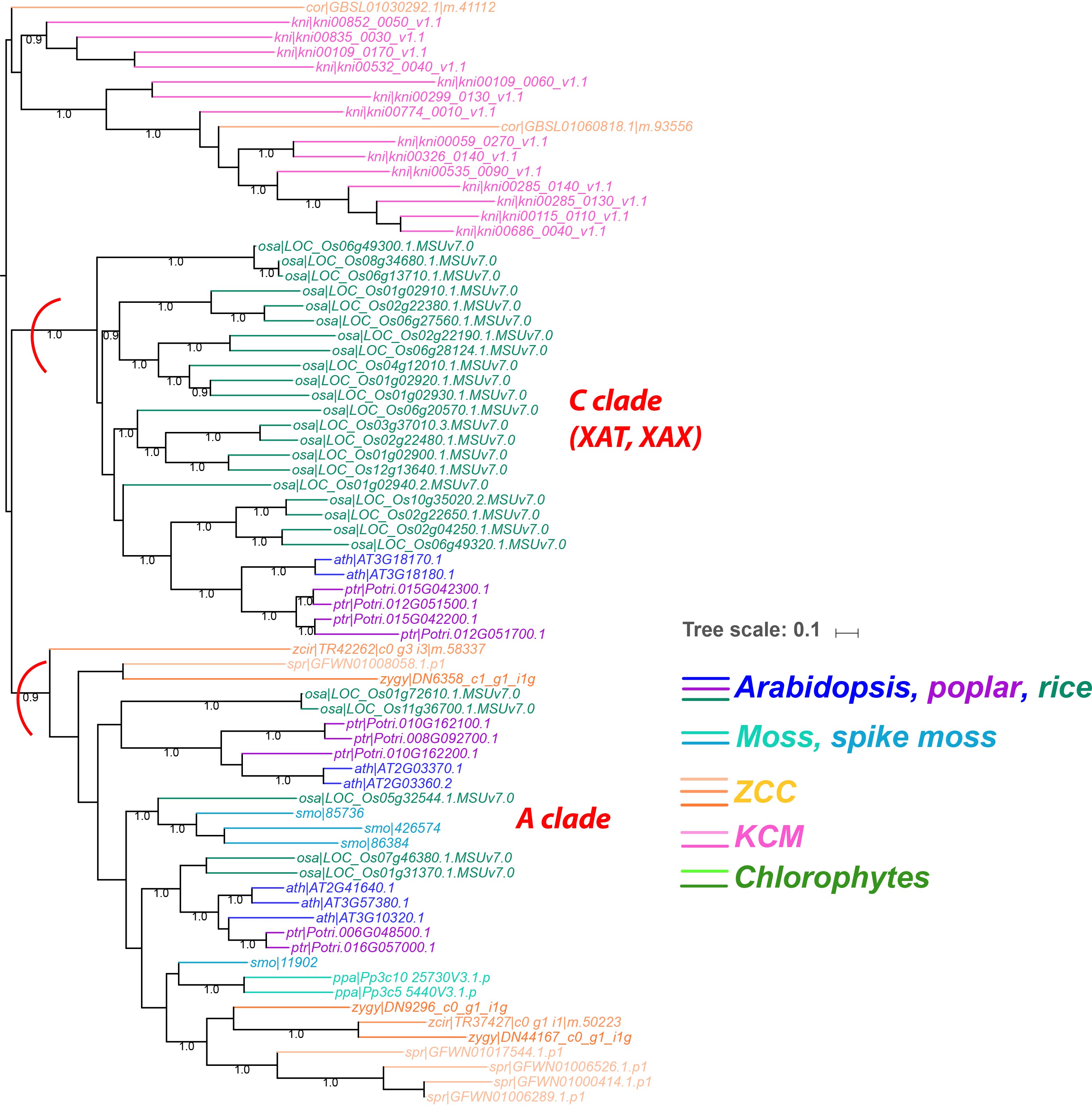

Supplement: FIGURE S4 — The phylogeny of GT61 proteins from selected species of land plants and algae. In total 74 GT61 protein sequences of 14 plant and algal species were used to build this phylogeny (see section “Materials and Methods”). The A and C clades classification was adopted from Chiniquy et al. (2012). The characterized grass XAT and XAX were indicated to be present in the clade C. [file Image_4.JPEG]

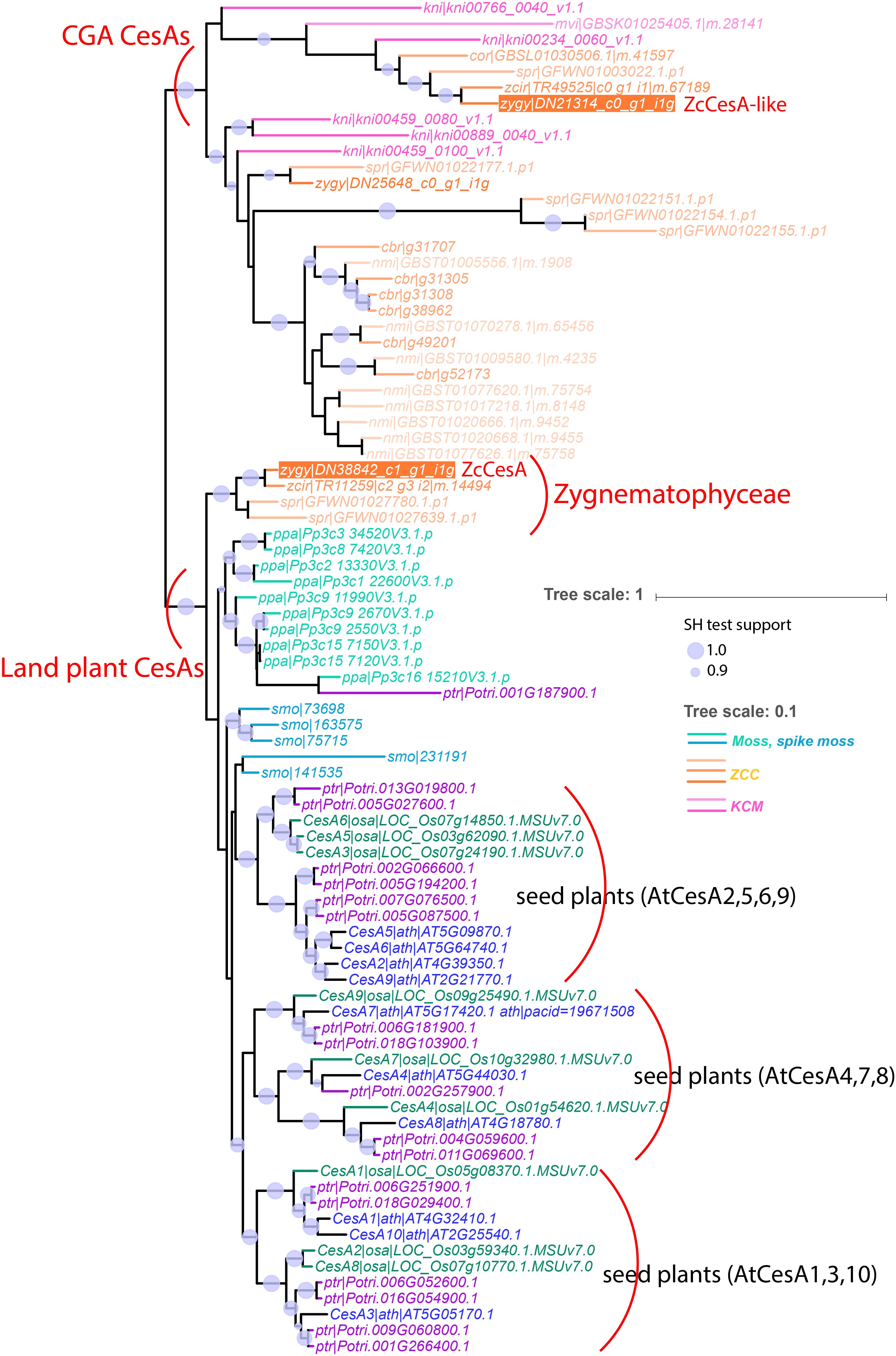

Supplement: FIGURE S5 — The phylogeny with land plant CesAs and CGA CesAs (former CslD-like clade). This is the complete version of Figure 5. [file Image_5.JPEG]
