# Supplementary material for: Cell Wall Enzymes in Zygnema circumcarinatum UTEX 1559 Respond to Osmotic Stress in a Plant-Like Fashion
Source: Front Plant Sci. 2019 Jun 7;10:732. doi: 10.3389/fpls.2019.00732 (PMC6566377; doi:10.3389/fpls.2019.00732)
Supplement: TABLE S1 — qRT-PCR primers of the 15 GT candidate genes. [file Table_1.DOCX]

**Table S1: qRT-PCR primers of the 15 GT candidate genes**

| Gene name | Left Primer 5’-3’ | Right Primer 5’-3’ |
| --- | --- | --- |
| ZcCesA | AGTGAATTTGCTCGCCGCTGGGTTCCGTTC | CGACGCTCCTTGACGAAAGTTGGCTGCACCT |
| ZcCesA-like | AACTGCAACGGACATGGAACCCCCGCCAAG | TTCCCCCAATCGCTGTTGTCTTCATAGTCG |
| ZcCslC | GCCGAAGGATGTGAAGGAAATTGGGAGGAGTCGAA | GCAAAGTCATAAAGCCGAGCCCGACCCATGC |
| ZcCslA-like | CTGCTGCCCAGTTGCGCCCTCTTCATCAGT | TGCCTCTTCAACGACCTCAGCCTCCCTGCTG |
| ZcGAUT3 | GATCGAAACAGCTAACCGCATTGACAGAGTC | TCACTCGCACAACCTGATCCTCATTCTCATCG |
| ZcGAUT10 | TCGTCACAGCCGCCAGTTTTTCGCAAGAGA | GTGTCGCACGGACAGCAGACTCCATTTCG |
| ZcGAUT13 | TGAGAGGGAACGAGTGACCATGCTTCAACG | CCGGCTCAGGAAGTGGCTTTTGTGCTTGTG |
| ZcGATL7-like | GGACCGCGAGCCCTTCACACAGGATGAGAC | GGACGAGTTTCGGAGCAACAGGCCCGACAG |
| ZcGolS | TGCTTGCGGAGTGTTTTGTCTGTCTCCCGAATG | CCTTTGTGGGGTCAAATCGCCAGGGTTTCG |
| ZcPGSIP-A-like | GTCCAGCAGGTCATCCCACGCTCTGCACAC | TGCTTGCATCTCCCAAGTGCTCCGCTGCAT |
| ZcPGSIP-B | TGCTTTCTTGCTTGTGACCACTTCAGCGAATGC | GCGGCCTCCTCTGTGACTCCCTTTGACACCA |
| ZcPGSIP-C | TCCCTCTCCTTACTCCCTGGCTTGGCCTCA | TCATGTGACCACCCCTTCGCCATCAGAGTC |
| ZcGT43-A | GCGCTGCTAAGGCGCTGAATGTTGATCTGG | AGGGGAGGAGGAGGAAGGAGGGAGCGTTGC |
| ZcGT43-B | GCGAGCCCCCTTGCTTTGGGTTGTGATTGA | GCCACCTTTCCTCCCACGCCTCTGGCATTT |
| ZcGT43-C | AGTCCTCTCCCCTTGCAGCCTCTCCCTGTC | TCCTTTTTCAGCCGCGATTTTTGGGCAGTC |
